# Changes in Cationic Selectivity of the Nicotinic Channel at the Rat Ganglionic Synapse: A Role for Chloride Ions?

**DOI:** 10.1371/journal.pone.0017318

**Published:** 2011-02-25

**Authors:** Oscar Sacchi, Maria Lisa Rossi, Rita Canella, Riccardo Fesce

**Affiliations:** 1 Department of Biology and Evolution, Section of Physiology and Biophysics, Ferrara University, Ferrara, Italy; 2 Center of Neuroscience and DBSF, Insubria University, Varese, Italy; Mount Sinai School of Medicine, United States of America

## Abstract

The permeability of the nicotinic channel (nAChR) at the ganglionic synapse has been examined, in the intact rat superior cervical ganglion in vitro, by fitting the Goldman current equation to the synaptic current (EPSC) I–V relationship. Subsynaptic nAChRs, activated by neurally-released acetylcholine (ACh), were thus analyzed in an intact environment as natively expressed by the mature sympathetic neuron. Postsynaptic neuron hyperpolarization (from −40 to −90 mV) resulted in a change of the synaptic potassium/sodium permeability ratio (P_K_/P_Na_) from 1.40 to 0.92, corresponding to a reversible shift of the apparent acetylcholine equilibrium potential, E_ACh_, by about +10 mV. The effect was accompanied by a decrease of the peak synaptic conductance (g_syn_) and of the EPSC decay time constant. Reduction of [Cl^−^]_o_ to 18 mM resulted in a change of P_K_/P_Na_ from 1.57 (control) to 2.26, associated with a reversible shift of E_ACh_ by about −10 mV. Application of 200 nM αBgTx evoked P_K_/P_Na_ and g_syn_ modifications similar to those observed in reduced [Cl^−^]_o_. The two treatments were overlapping and complementary, as if the same site/mechanism were involved. The difference current before and after chloride reduction or toxin application exhibited a strongly positive equilibrium potential, which could not be explained by the block of a calcium component of the EPSC. Observations under current-clamp conditions suggest that the driving force modification of the EPSC due to P_K_/P_Na_ changes represent an additional powerful integrative mechanism of neuron behavior. A possible role for chloride ions is suggested: the nAChR selectivity was actually reduced by increased chloride gradient (membrane hyperpolarization), while it was increased, moving towards a channel preferentially permeable for potassium, when the chloride gradient was reduced.

## Introduction

Nicotinic receptors (nAChR) are perhaps the most thoroughly characterized family of ligand-gated ion channels. The muscular subtypes are easily studied at the intact neuromuscular preparation, and the differential properties of the mature junctional nAChR vs. the extrajunctional or embryonic subtype have been precisely defined. In contrast, neuronal nAChRs occur in a variety of subtypes and information on their selective localization, at the postsynaptic site or in other regions of the neuronal plasmalemma, are scarce and contradictory. Similarly ill-defined are the biophysical properties of the native nAChR subtypes, and whether differences may occur between the subsynaptic and extrasynaptic receptors in terms of response to acetylcholine and agonist or antagonist compounds.

The precise structural motifs that determine the relative permeability to the various cation species have been intensely studied. The first report of a nicotinic channel with modified permeability regarded a mutated α7 homomeric nAChR [1]. Subsequently, point mutations of single subunits were shown to be able to decrease or increase cation selectivity of nAChR channels (see, for example, reviews by Skok [2] and Keramidas et al. [3]). In those experiments, mostly performed by heterologous expression of cloned receptors, a structural modification had to be produced in the channel protein to induce significant changes in selectivity.

The nAChR is also known to be regulated by protein phosphorylation of its cytoplasmic domains by protein kinases, which phosphorylate this receptor at distinct sites [4]; however, phosphorylation altered the kinetics of receptor desensitization, but in no case it appeared to affect the permeation properties of the channel [5], [6], [7], [8].

In general, little is known about possible changes in neuronal channel selectivity among cations, and none of the usual experimental approaches could reveal anything about the important question whether subsynaptic or extrasynaptic nAChRs were different functional entities.

We recently reported that the properties of the subsynaptic native nAChRs, in response to the physiologically released ACh, are modified within a few hours after denervation of the rat postganglionic neuron [9]. In particular, the current-voltage relations for EPSCs indicated a change in nAChR ion selectivity, suggestive of a switch from a cationic channel preferably permeable to potassium ions to a pore with no selectivity between the two ions; the extrasynaptic receptor properties were insensitive to denervation. Those observations warned about the importance of selecting the appropriate subpopulation of receptors, in order to be able to extract information relevant to physiology; on the other hand, they pointed out an unexpected flexibility of the nicotinic channel in its permeation properties.

Here we report the analysis of simple experimental conditions, which are unable to acutely produce any change in nAChR subunit composition. Shifts of the resting membrane potential within a voltage range of physiological interest, ionic modifications and the action of specific toxins, however, are shown to be accompanied in intact sympathetic ganglia by changes of the nAChR selectivity, which may significantly control the efficiency of synaptic transmission. On the other hand, the molecular mechanisms by which the binding of a toxin, or simple changes in chloride concentrations, induce such functional changes remain elusive.

## Methods

### General procedures

Electrophysiological experiments were performed on superior cervical ganglia isolated from rats (120–250 g body weight) during urethane anesthesia (1–1.5 g kg^−1^; I.P. injection) and maintained in vitro at 37°C. After surgery, the animals were killed with an overdose of anesthetic. The use and handling of animals was approved by the Animal Care and Use Committee of the Ferrara University (approval ID: 11214 of May 16, 2006). The ganglion, dissected together with the sympathetic trunk, was desheathed and pinned to the bottom of a chamber mounted on the stage of a compound microscope; individual neurons were identified at a magnification of ×500 by using diffraction interference optics.

### Solutions and drugs

The preparation was continuously superfused with a medium (mM: 136 NaCl, 5.6 KCl, 5 CaCl_2_, 1.2 MgCl_2_, 1.2 NaH_2_PO_4_, 14.3 NaHCO_3_, 5.5 glucose) pregassed with 95% O_2_-5% CO_2_ to a final pH 7.3. Atropine sulfate (Sigma) 10^−6^M was systematically added to the saline. The nicotinic antagonists α-bungarotoxin (αBgTx, Sigma), N,N,N-trimethyl-1-(4-trans-stilbenoxy)-2-propylammonium iodide (F3) and methyllycaconitine citrate (MLA, Tocris) were bath-applied by exchanging the normal saline with drug-containing medium by means of a continuous rapid perfusion system.

### Electrophysiological recording and data acquisition

Neurons were impaled with two independent glass microelectrodes filled with neutralized 4 M potassium acetate (30–40 MΩ resistance). Recordings were obtained under two-electrode voltage-clamp conditions as described previously [10] using a custom-made amplifier. The bath was grounded through an agar-3 M KCl bridge.

[Cl^−^]_o_ was reduced by substituting 136 mM Na-isethionate or Na-benzenesulfonate for an isoosmolar amount of NaCl. The low-chloride solutions were applied when both microelectrodes were inside the neuron.

Impalement of the cell produces a change in liquid junction potential at the electrode-solution interface which can be estimated, under the present extra- and intracellular ionic composition, to −2.46 mV. This correction should be applied to all measurements. Furthermore, changes in intracellular and/or extracellular chloride ions are expected to introduce shifts in such diffusion potentials, respectively, at the microelectrode and reference electrode interfaces. Considering all the conditions here examined, the corrections which would be applied to measured membrane potential hardly varied among the various conditions (−2.2 to −2.5 mV, 4 M K-acetate microelectrode), except low external chloride (+0.04 mV). The variations were too small to interfere with the reported effects, so the uncorrected measurements are reported in the results (see Text S1 for the corrected values and a detailed computation of liquid junction potentials at the microelectrode and reference agar bridge).

Potentials arising between the bath and the reference agar-bridge electrode were measured by comparing the potential of the fresh 3 M KCl agar bridge with that of a broken-tip microelectrode filled with 3 M KCl [11]. Values of +1.1±0.4 mV after 5 min and of +2.4±0.3 mV after 10 min were measured following isethionate replacement (n = 8), and of −0.9±0.3 mV in the presence of benzenesulfonate (+1.4±0.2 mV after an additional 10 min wash in control saline; n = 6). A 5 min perfusion period with the modified solution was usually sufficient to perform complete liquid substitution in the chamber and the electrophysiological tests. The voltage shifts measured by the 3 M KCl microelectrode thus suggest that the liquid junction potential at the salt bridge was stable and too small to affect our results. Two further checks were performed to exclude that chloride substitution might introduce artifacts in estimates of membrane potential. The EPSC I–V relationship was evaluated in two experiments, in which microelectrodes filled with 3 M KCl (instead of K-acetate) and a reference electrode with a stable half-cell potential essentially independent of electrolyte concentration (Super-Dri-Ref SDR2, WPI) were used. Finally, a functional test was performed by measuring the peak amplitude of the delayed potassium current in the −30/−10 mV range, over which the voltage dependence of the I–V curve becomes steep. In this voltage region, in fact, about 8 mV are sufficient to change by e-fold the steady-state activation variable n_∞_ (according to our previous Hodgkin-Huxley-type analysis of the ganglionic delayed potassium current). When [Cl^−^]_o_ was modified the potassium current amplitudes were found unchanged (31.2±2.3 vs. 32.5±2.9 nA at −10 mV; n = 5). The portion of the K^+^ I–V curve above −30 mV was thus insensitive to external chloride reduction, ruling out both the presence of any systematic voltage shift due to external anionic modification, and marked changes in internal K^+^ concentration.

In order to activate the preganglionic input, single supramaximal current pulses of 0.3 ms duration were applied to the cervical sympathetic trunk through a fine suction electrode, positioned close to the caudal pole of the ganglion. Each I–V curve was built by applying a cycle of command voltage steps (0.1 Hz, 200 ms duration, test potential in the −30/−100 mV range) to the post-synaptic membrane and delivering one preganglionic pulse 30–40 ms after the onset of each voltage step. The EPSC ensued and developed its whole time course when any other ionic current, possibly activated by the voltage step, should have settled to the new level corresponding to the command voltage. The only rapidly transient current, which might contaminate the EPSC time course, when the holding potential was −90 mV, is IA; however, the time interval between the voltage step onset and ACh release was sufficiently long to allow complete IA inactivation by the time the EPSC was evoked. After each step, the postsynaptic membrane potential was returned to −50 mV, or to a different holding potential when specified. Large synaptic currents could be recorded with good control of the membrane potential at any tested voltage [15]. From the EPSCs recorded at the different command potentials the I–V relationship was derived and the ACh reversal potential (E_ACh_) that prevailed over the range of physiological membrane potential values here considered (−30/−100 mV) was estimated by extrapolating the Goldman current equation fitted to the whole data set (see below).

Normal saline made hypertonic by the addition of 0.37 M sucrose (final osmolality 718 mosm/kg), or K^+^-enriched solutions (final [K^+^]_o_ = 35 mM) were used to increase the presynaptic release of quantal events. Long-lasting tracings containing randomly occurring miniature potentials (mEPSPs) were recorded continuously on a digital recorder (Biologic, DTR-1200; 0–10 kHz) under two-electrode current-clamp conditions; the post-synaptic membrane potential was held at −50 mV by passing current through the current electrode and switched to different steady levels, in random sequence, for periods of 20–60 s, separated by at least 2 min rest at −50 mV. Subsequently, the recordings were sampled at 10 kHz and analysed off-line.

Tracings were filtered at 5 kHz with an 8-pole Bessel filter, digitized at 10 kHz with a 12-bit analog-to-digital interface (Digidata 1200A operated by pCLAMP software, Axon Instruments) and stored on disk for future analysis with pCLAMP (version 5.5; Axon Instruments, Union City, CA) and MATLAB 5.0 (The MathWorks, Natick, MA) software packages.

### General data analysis

The relative permeability of the cations carrying the synaptic current is usually evaluated by using the Goldman equation and measuring the shift of the current reversal potential following changes of single ion concentrations. Considering the possible inaccuracy in the evaluation of the permeability ratios from a single extrapolated value, we have preferred to examine the permeability of the ions generating the macroscopic EPSC by fitting the Goldman equation to the measured synaptic current-voltage relations over the entire voltage range available (usually the −30/−100 mV range). We have recently shown that the calcium permeability does not contribute significantly to the synaptic reversal potential in the range 0.1–8 mM external Ca^2+^, and that the calcium current fraction in the EPSC genesis is quantitatively too small to affect, by omitting it, any of the derived conclusions [9]. Thus, the EPSC has been considered as I_Syn_ = I_Na_+I_K_, and the current carried by each single monovalent cation (“x”) given by the equation: 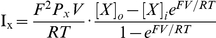, with P_x_ indicating the permeability of ion “x”.

Experimental data have been fitted by minimizing the sum of the square of the errors to provide the absolute value of potassium (P_K_) and sodium permeability (P_Na_) for synaptic macrocurrents; the resulting P_K_/P_Na_ ratio, which is independent of the number of channels and size of the cell, was computed and compared among the various experimental conditions. The corresponding virtual equilibrium potential, E_ACh_, is also reported as it may be more directly informative. The nicotinic current evoked by the native ACh in neurons of intact amphibian [12], [13] or mammalian [14], [15] sympathetic ganglia exhibits rectification only at positive membrane potentials. However, we did not explore membrane potentials positive to −20 mV because in that region delayed potassium currents significantly interfere with the measurements. Actually, the I–V curves are perfectly fit by the Goldman current equation in the −100/−30 mV range using only two free parameters (total cell nAChR conductance for Na^+^ and for K^+^) and what we refer to as virtual E_ACh_ merely is a value mathematically derived from P_K_/P_Na_, which is not affected by possible departures of the channel behavior from the Goldman model at more positive potentials. Constant [Na^+^]_i_ = 32 mM and [K^+^]_i_ = 190 mM were assumed (see Discussion).

The differences among experimental conditions were examined by one or two-way ANOVA. Values of F and *P* are reported in the text for treatment effect. In the figures, data are reported by pooling the results obtained from several cells under each experimental condition. Average values and SEM are plotted for each condition.

## Results

The intact and mature rat sympathetic neuron in vitro is used to analyze the basic effects of the naturally released ACh. The good voltage control of the postsynaptic neuron, typically obtained with the two-microelectrode technique, provides reliable EPSC I–V curves, which make possible to perform a biophysical analysis of the permeation properties of the subsynaptic channels that generate the macroscopic synaptic current. The relatively slow changes (over several minutes) produced in cation selectivity by simple treatments, such as membrane potential migration, changes in external chloride composition and αBgTx application, in the absence of any structural channel modification, challenge the conventional view of a stable behavior of the synaptic channel ionic selectivity.

### Effect of membrane potential on EPSC properties

**Figure 1:**
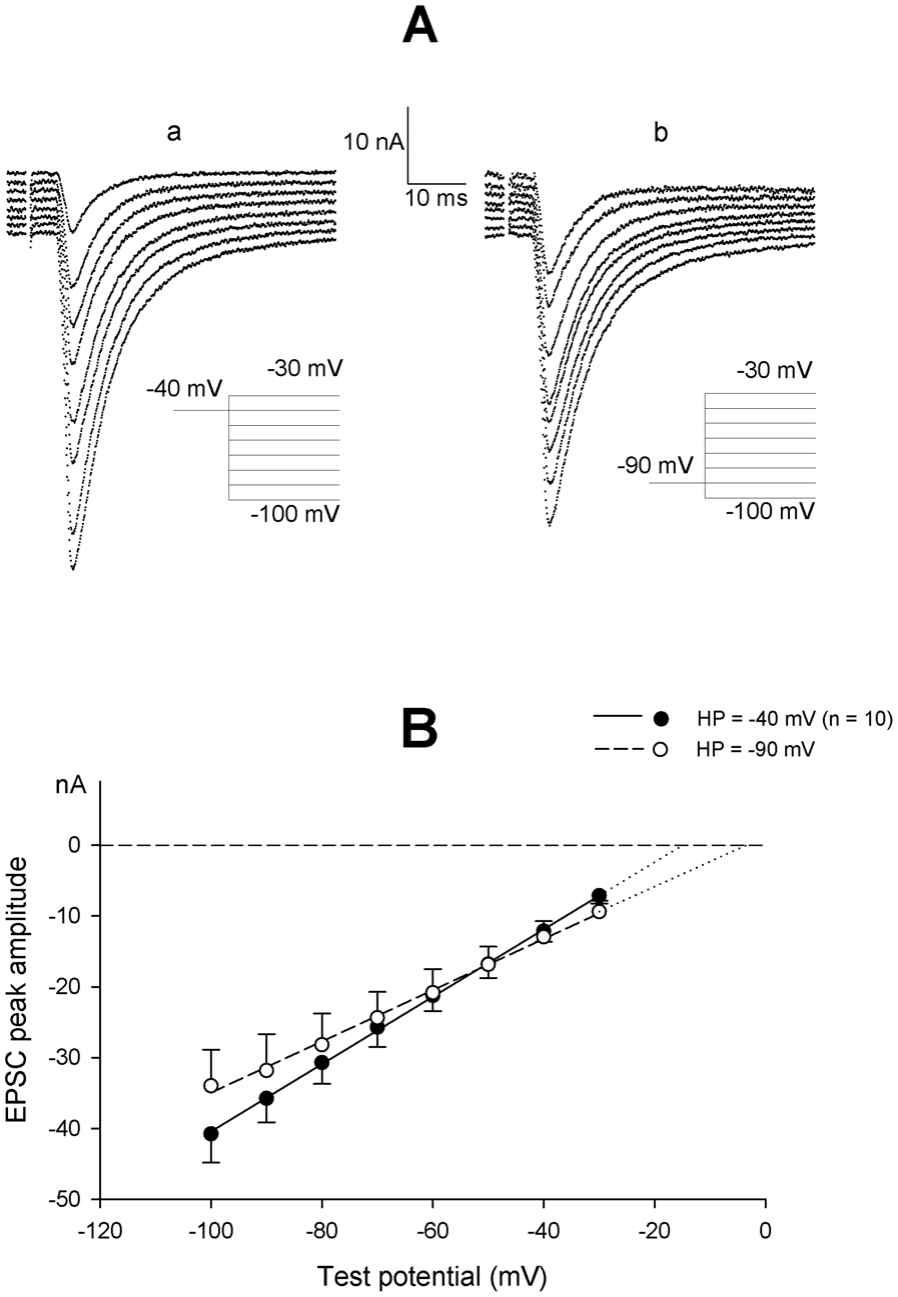
Effect of holding potential on EPSC properties. (**A**). Representative tracings showing the effect of voltage on the EPSC I–V relationship in a neuron maintained at −40 (a) or −90 mV (b) holding potential. EPSCs were evoked in either case at test command potentials over the same −30/−100 mV membrane potential range in 10 mV steps. Peak EPSC amplitude of the tracings was fitted by the Goldman current equation over the whole voltage range tested, providing an estimate of E_ACh_ as derived from the P_K_/P_Na_ ratio (−14.5 mV for VH-40 and +3.1 mV for VH-90). (**B**). Mean I–V relationship for peak EPSC amplitude. Data from 10 neurons held at −40 (filled circles) and subsequently at −90 mV (open circles) holding potential. Analysis indicates a shift of the mean P_K_/P_Na_ ratio from 1.40±0.13 (VH-40) to 0.92±0.10 (VH-90).

The I–V curve of EPSCs elicited over the −30/−100 mV voltage range has been measured in neurons held at −40 mV (VH-40); each neuron was thereafter brought to and held at −90 mV for at least 3 min (VH-90), and the pulse sequence was repeated from this new holding level; a third trial was performed after returning to −40 mV (VH-40R). Typical EPSC families recorded in the same neuron, starting from the two holding potentials, are illustrated in Figure 1A; the mean peak EPSC amplitude measured in a 10-neuron sample is plotted in Figure 1B against the membrane level at which synaptic currents were evoked. Both EPSC amplitude and relative slope of the I–V curve were affected by increased membrane negativity: the P_K_/P_Na_ ratio decreased from 1.40±0.13 at VH-40 to 0.92±0.10 at VH-90, the mean virtual E_ACh_ shifted from −15.7±1.0 mV to −5.9±2.0 mV, while the mean synaptic conductance, g_syn_ (evaluated from the slope of the I–V relationship), decreased from 0.48±0.4 µS to 0.38±0.5 µS. The entire −40/−90/−40 mV cycle was tested in 5 neurons to demonstrate that the voltage effects were reversible after the steady-state was regained at the starting membrane potential (−14.3±1.7, −4.2±3.8, −16.2±1.6 mV for the E_ACh_ at VH-40, VH-90 and VH-40R, respectively; 0.53±0.06, 0.44±0.01, 0.53±0.08 µS for g_syn_).

The modifications in P_K_/P_Na_ ratio were quite slow: minutes were required for its settlement, at any new holding potential. The shifts in the corresponding virtual E_ACh_ followed an exponential time course upon depolarization to −40 mV (mean time constant of 3.2 min, n = 5), and were virtually complete within 4–5 min when the neuron was hyperpolarized to −90 mV.

**Figure 2:**
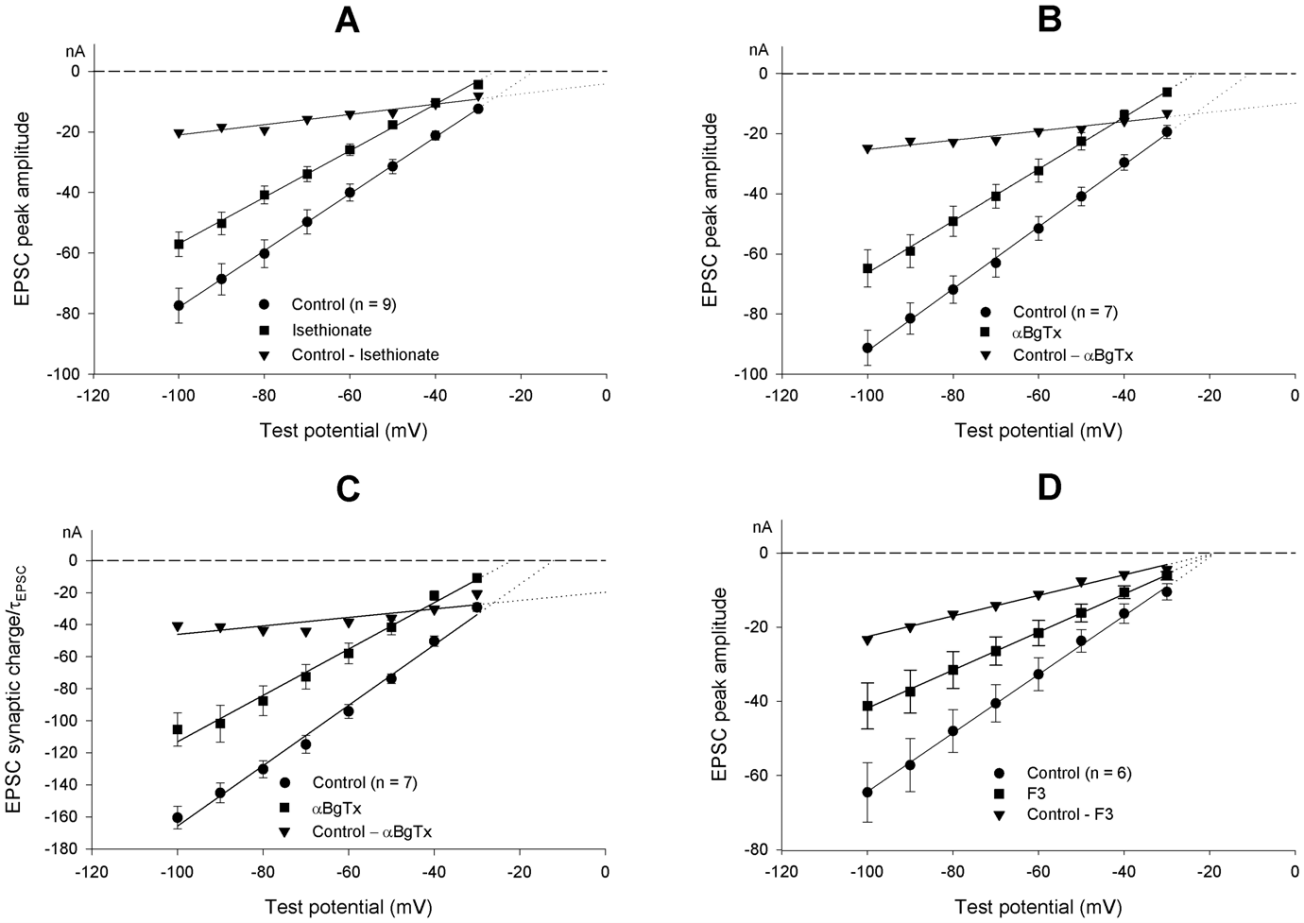
Effect of [Cl^**−**^]_o_ modifications and of pharmacological treatments on EPSC I–V curves. (**A**). Mean I–V relationship of EPSCs evoked before (circles) and after substitution of 136 mM isethionate for an isoosmotic amount of NaCl (squares). Analysis indicates a shift of the P_K_/P_Na_ ratio from 1.57±0.09 to 2.26±0.08 in low chloride solution. Peak amplitude difference values (triangles) are used to build the I–V curve of the current cancelled by the treatment: note its largely positive equilibrium potential. (**B**). Effect of 200 nM αBgTx application. The I–V curves of the EPSC peak amplitude before (circles) and after toxin treatment (squares) are shown. The difference current relationship (triangles) was drawn as in (**A**). (**C**). The ratio EPSC-charge/EPSC-decay time constant is fitted against test potential (same data as in **B**). (**D**). Effect of the selective nicotinic antagonist F3 (10 µM; squares) vs. controls (circles); triangles show the corresponding difference plot of the EPSC amplitudes.

The brief conduction time in preganglionic fibers results in a quasi synchronous ACh release at the presynaptic terminals following stimulation, so that the EPSC time course is strictly related to the interaction of ACh with the nicotinic receptors. The synaptic current onset was not sensitive to the postganglionic membrane potential (either holding or test potentials) and the EPSC time-to-peak remained remarkably constant at 2.1±0.1 ms (n = 11). The synaptic current decay was described by a single exponential function of time with a mild voltage dependence on command voltage. The EPSC decay time constant, but not its voltage dependence on the command potential, was sensitive to the holding potential. The average decay constant significantly decreased when currents were elicited from hyperpolarized neurons: the mean time constants in neurons held at −40 mV were 6.3±0.5 and 8.2±0.6 ms for −40 mV and −90 mV command potentials, respectively, whereas in neurons held at −90 mV the corresponding values were 4.8±0.4 and 6.4±0.5 ms (n = 10). Since the decay time constant reflects the mean open time of the nicotinic channel, its modifications affect the overall synaptic charge. In fact, while the peak synaptic current, given the existing density of receptors, mostly depends on the magnitude of synchronous quantal emission and the driving force for the permeable ion species, the total synaptic charge is also affected by the time the nicotinic channel remains open. This offers an alternative approach to estimate the driving force and E_ACh_. The current through the receptor was thus evaluated from the same current tracings as in Figure 1B by computing the ratio synaptic-charge/τ_EPSC_ and plotted versus the test potential. These new null point estimates (np_-40mV_ = −17.6 mV; np_-90mV_ = +0.4 mV) were very similar to those extracted from the EPSC peak amplitudes in Figure 1B. The voltage effect on the synaptic-charge/voltage curve proved to be fully reversible (n = 4). A similar analysis is applied in Figure 2C to a different neuron sample.

Fitting of the series of I–V curves obtained in VH-40 neurons provided the following mean values of absolute permeability per neuron: P_K_ = 0.984 fl/s and P_Na_ = 0.701 fl/s, suggesting a permeability ratio P_K_/P_Na_ = 1.40±0.13 (n = 10). The observed changes in the I–V curves and shift in null potential in the same neurons held at VH-90 would be accounted for by a change of the P_K_/P_Na_ value to 0.92±0.10 (P_K_ = 0.582 fl/s and P_Na_ = 0.632 fl/s). Modifications due to voltage would thus mainly involve the potassium channel permeability.

Statistical analysis confirmed that the P_K_/P_Na_ estimates evaluated in single neurons, at VH-40 *vs*. VH-90, were significantly different (*P*<0.01, paired Student's *t*-test). A similar difference was observed in the mean E_ACh_ values (*P*<0.01, paired Student's *t*-test) in neurons maintained at the two holding potentials. Two-way ANOVA demonstrated that the EPSC decay time constant values changed significantly from VH-40 to VH-90 (*F* = 48.8, *P*<0.01) and displayed a significant voltage dependence (*F* = 12.2, *P*<0.01), which did not appear to change in the two conditions (*F* = 0.1).

### Effects of [Cl^−^]_o_ modifications

The very slow time course of the observed voltage-dependent changes in channel properties suggests that the action of the holding potential may be mediated by some slow processes. In the sympathetic neuron, modifications in membrane potential level are accompanied by a chloride ion redistribution, which develops with a time constant in the 20–40 s range [16], and may therefore constitute a good candidate as a process that drives the observed slow changes in AChR properties. In sympathetic ganglia, the reversal potential of the nicotinic current is considered to depend on the transmembrane gradient of sodium and potassium ions, and to be insensitive to chloride ions [17], [18]. Thus, we have re-examined the possibility that chloride might interfere with nAChR channel operation, by changing external chloride concentration. Data were obtained in 10 neurons after substituting 136 mM of either Na-isethionate (n = 7) or Na-benzenesulfonate for an isoosmotic amount of NaCl. Results were similar and are cumulatively presented in Figure 2A. The ionic manipulation systematically decreased EPSC amplitudes and slopes of the I–V curves, generated a −9.7±1.3 mV shift in virtual E_ACh_ and a 19.2±2.3% decrease in g_syn_ (*P*<0.001 for differences in both data groups, paired Student's *t*-test). These effects were reversible upon washing (n = 5). The time-to-peak of the synaptic current was unaffected by this ionic treatment, while the EPSC decay time constant proved to be slightly but significantly reduced by the decreased [Cl^−^]_o_ (−13% at −70 mV and −11% at −90 mV; *P*<0.01 for both data groups, paired Student's *t*-test).

The effects observed after chloride substitution might be contributed to by a change in Ca^2+^ activity, due to Ca^2+^ binding to the substituting anion. Isethionate, but not benzenesulphonate, actually exhibits some Ca^2+^ binding activity [19]. A presynaptic effect, i.e. reduced Ca^2+^-dependent quantal emission, might sustain the decreased EPSC amplitude shown in Figure 2A, but not the associated selectivity change of the synaptic channel (see below).

When [Cl^−^]_o_ was reduced to 18 mM, the typical dependence of E_ACh_ on holding potential, described in the preceding paragraphs, was cancelled. In 4 neurons exposed to 136 mM Na-isethionate the E_ACh_ was −22.8±3.9 mV at VH-40 and −23.5±3.8 mV at VH-90 (as opposed to a VH-40/VH-90 shift of about 10 mV in normal saline).

The EPSC amplitude decrease, systematically observed in chloride-deprived solutions, suggests that chloride omission may cancel a component of the synaptic current. When the difference in EPSC amplitude, before and after ionic modification, is plotted *vs*. membrane potential, a strongly positive null potential (+24.0 mV) is obtained for this difference current (Fig. 2A). In principle, this result might be due to an ill-defined participation of the chloride current to the overall synaptic current, or to a modification of the permeability of the nicotinic channel. We utilized the Goldman model, initially based on the pure cationic nature of the EPSC, to examine the unconventional possibility of EPSC being carried also by a chloride current component (EPSC = I_Na_+I_K_+I_Cl_). Under this hypothesis, the equation describing the current carried by monovalent ion species X can be rewritten as 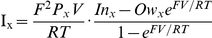 where 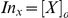 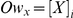 for a cation, and 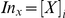, 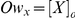 for chloride ion; the EPSC is predicted by the equation 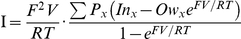, and the difference current, ΔI, between the EPSC in normal Ringer's and the EPSC in {0-Cl}, is simply given by 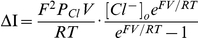. This formula necessarily (and obviously) yields a positive value, i.e. an outward current (it corresponds to the Cl^–^ inflow that has been abolished). Actually, the difference current was negative (Fig. 2A), and fitting this hypothesis would lead to a negative estimate of *P_Cl_*≈–0.2 pl/s for nAChRs. We therefore definitively discarded the hypothesis of chloride permeation through the nAChR channel.

The synaptic current was thus considered as cationic in nature, with the important complement that [Cl^−^]_o_/[Cl^−^]_i_ controls the permeability and ion selectivity of the nicotinic channel. The numerical solution of the fit of the experimental data, based on this assumption, is that the P_K_/P_Na_ ratio = 1.57±0.09 with [Cl^−^]_o_ = 154 mM (P_K_ = 2.162 fl/s and P_Na_ = 1.361 fl/s) becomes 2.26±0.08 with [Cl^−^]_o_ = 18 mM (P_K_ = 2.229 fl/s and P_Na_ = 0.977 fl/s; n = 10). These P_K_/P_Na_ estimates are significantly different (*P*<0.001, paired Student's *t*-test). The [Cl^−^]_o_ –dependent changes of the synaptic channel would thus mainly involve the sodium permeability.

### EPSC properties after pharmacological treatments

The synaptic macrocurrent in the sympathetic ganglion may arise from a mix of different nicotinic channels, with different subunit composition and functional properties [20]. The shift of the E_ACh_ toward a negative membrane potential following ionic modification might arise from a selection among them, due to differential sensitivity to the ionic composition. The rat sympathetic neurons actually express an αBgTx-sensitive nAChR which is likely to incorporate the α7 subunit to yield channels of unusually high Ca^2+^ permeability. This receptor type might carry a current with a positive null potential, that, when cancelled, might justify a negative shift of the EPSC null potential.

We have tested the effect of 200 nM αBgTx at the ganglionic synapse. Data obtained from 7 neurons are illustrated in Figure 2B. Toxin application (for at least 5 min, while the neuron was maintained at −50 mV holding potential) resulted in an evident decrease of the EPSC amplitudes accompanied by modification of the slope of the EPSC I–V curve; the mean E_ACh_ changed by −12.6±2.8 mV and g_syn_ by −12.8±3.1%. These estimates were confirmed when the EPSC synaptic-charge/τ_EPSC_ parameter in the same neurons was plotted versus the test potential (Fig. 2C). The EPSC decay remained monoexponential in the presence of the toxin; the decay time constant was significantly reduced compared with controls (−23.2% at −40 mV; −16.1% at −70 mV), by amounts similar to those observed in the low [Cl^−^]_o_ experiments.

The I–V of the difference current, and synaptic charge, before and after αBgTx treatment exhibited a reduced slope (Fig. 2B, triangles) suggesting a largely positive null point (+63.3 to +75.3 mV), as if a current fraction with a strongly positive equilibrium potential were blocked by the toxin. To examine the possibility that αBgTx selectively blocked a subpopulation of channels, other blockers were tested.

The 4-oxystilbene derivate F3 has a high selectivity for neuronal nicotinic αBgTx receptors containing the α7 subunit, in chick, and low activity against brain-type nAChRs [21]. In mammals it is also active on non-α7-containing receptors, but it retains selectivity for α7-containing receptors (IC_50_≈1 nM for rat homomeric α7 receptors expressed in *Xenopus* oocytes) [22]. The data obtained from 6 neurons exposed to 10 µM F3 are illustrated in Figure 2D. The drug reduced the mean g_syn_ by 36% without any effect on the E_ACh_, suggesting that a subpopulation of channels is presumably blocked, but unblocked channels retain their normal selectivity properties. Similar results were obtained using a specific antagonist for α7-containing neuronal nicotinic receptors, methyllycaconitine (MLA): 10 nM MLA applied to the bath did not noticeably affect E_ACh_ (a mean difference of −0.9 mV; n = 4), while g_syn_ decreased by a statistically not significant 10.8%. These results rule out the hypothesis that the αBgTx action on the ganglionic synaptic current might arise from selective blockade of an α7-dependent calcium current fraction and point to a specific effect of αBgTx on the selectivity properties of the subsynaptic nAChR channel.

**Figure 3:**
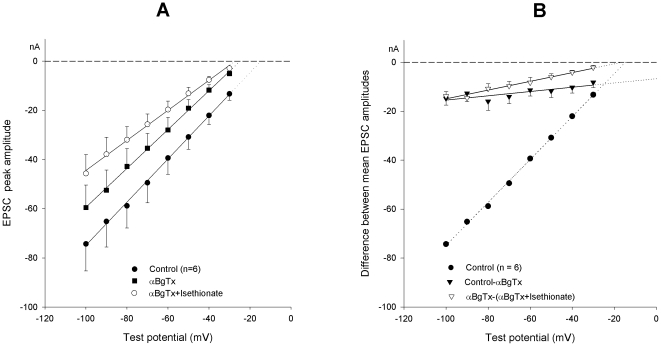
Cumulative effects of αBgTx application and [Cl^**−**^]_o_ reduction. (**A**). I–V relationship of EPSC peak amplitude in control (filled circles; P_K_/P_Na_ ratio  = 1.52±0.11), after 200 nM αBgTx application (squares; P_K_/P_Na_ ratio  = 2.22±0.13) and after cumulative isethionate replacement (open circles; P_K_/P_Na_ ratio  = 2.38±0.07), substituting for 136 mM NaCl. Goldman equation curves are fitted to the mean values from the same 6-neuron pool. (**B**). I–V curves of the difference currents showing the effect of αBgTx (filled triangles; note the largely positive equilibrium potential) and of the subsequent application of the low-chloride solution in the presence of the toxin (open triangles). Control values (circles) and the EPSC peak amplitudes generating the difference currents are the same as in (**A**). Zero crossing points of the fits indicate the corresponding E_ACh_ estimates.

Manipulation of [Cl^−^]_o_ or of the nicotinic receptor by αBgTx resulted in phenomenologically similar modifications of the synaptic current, since both treatments similarly affected both channel permeation and kinetics. We verified the possible interactions between the two treatments by applying sequentially and cumulatively, in either order, the chloride reduction (136 mM Na-isethionate substitution for NaCl) and 200 nM αBgTx. Data from 6 neurons, in which the isethionate-solution was followed by the αBgTx application, are presented in Figure 3 and Table 1. The same table also reports the results of the mirror experiment, in which the αBgTx treatment was followed by the [Cl^−^]_o_ reduction (n = 7). The effects appear to be partly overlapping and complementary. By comparing the data from the two experiments, the following conclusions can be drawn: 1) αBgTx or external chloride reduction modify the nicotinic cationic selectivity to approximately the same extent (P_K_/P_Na_ ratios move from 1.5 to about 2.2); 2) once the permeability properties of the channel are modified by either αBgTx or isethionate, the subsequent application of the second treatment (isethionate or αBgTx) does not further modify the channel properties significantly, indicating mutual occlusion of the effects; 3) the effects on g_syn_ are instead additive: external chloride reduction decreases the momentary available g_syn_ by the same amount (about −20%), independently of the application order; the same holds true for αBgTx (about −10%); 4) the ultimate cumulative effect (both in terms of cation selectivity and total conductance) is quantitatively the same, independent of the order in which the two treatments are applied.

**Table 1:** Modifications of E_ACh_, g_syn_ and the permeability ratio P_K_/P_Na_ after subsequent application, in either order, of 200 nM αBgTx and 136 mM isethionate (substituting for an isoosmotic amount of NaCl).

	Control (n = 6)	αBgTx	αBgTx+isethionate	Cumulative effect*
g_syn_	100%	Δ−9.5±2.6%	Δ−21.8±1.6%	Δ−29.2%
E_ACh_	−15.6±1.4 mV	Δ−9.5±2.4 mV	Δ−1.8±1.0 mV	Δ−11.3 mV
P_K_/P_Na_	1.52±0.11	2.22±0.13	2.38±0.07	

	Control (n = 7)	isethionate	isethionate+αBgTx	Cumulative effect*
g_syn_	100%	Δ−19.2±2.2%	Δ−11.8±3.8%	Δ−28.7%
E_ACh_	−16.2±1.3 mV	Δ−9.7±1.3 mV	Δ−1.9±1.4 mV	Δ−11.6 mV
P_K_/P_Na_	1.57±0.09	2.26±0.08	2.42±0.11	

*with respect to control.

Values are means ± SEM.

Statistical analysis (one-way ANOVA) of the P_K_/P_Na_ data presented in Table 1 confirmed the difference between groups (F = 18.12, *P*<0.01) and the marked increase in the permeability ratio (about 46%, 95%-CL = 27–64%) produced by αBgTx and thereafter by αBgTx plus isethionate (about 56%, 95%-CL = 38–74%). The difference between the two treatments did not reach statistical significance. Essentially the same statistical results were obtained when the order of treatment application was inverted and isethionate was applied first.

### mEPSPs under current-clamp conditions

**Figure 4:**
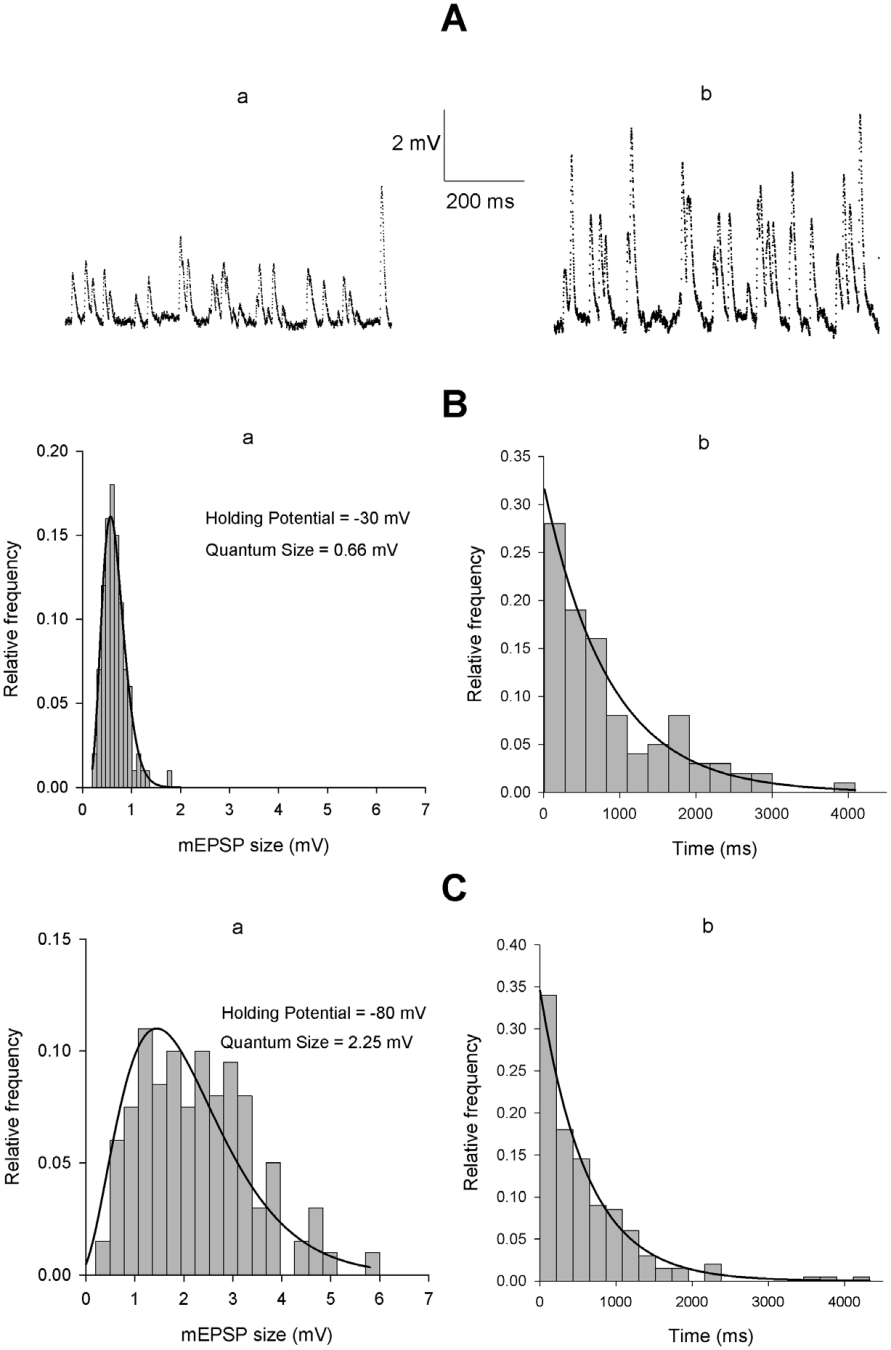
Properties of the mEPSPs elicited by 35 mM [K^**+**^]_o_ at different holding potentials. (**A**). Representative mEPSP recordings obtained under two-electrode current-clamp conditions in the same neuron at −35 mV (spontaneous) and −90 mV holding potential. (**B**) **and** (**C**). Examples of mEPSP amplitude (a) and time interval distributions (b) obtained in a different neuron at −30 and −80 mV holding potential. Amplitude histograms are fitted by a lognormal distribution; the interval distribution by an exponential function. Quantum size at each holding level is calculated as the arithmetic mean of the mEPSPs recorded during 20–60 s time periods.

**Figure 5:**
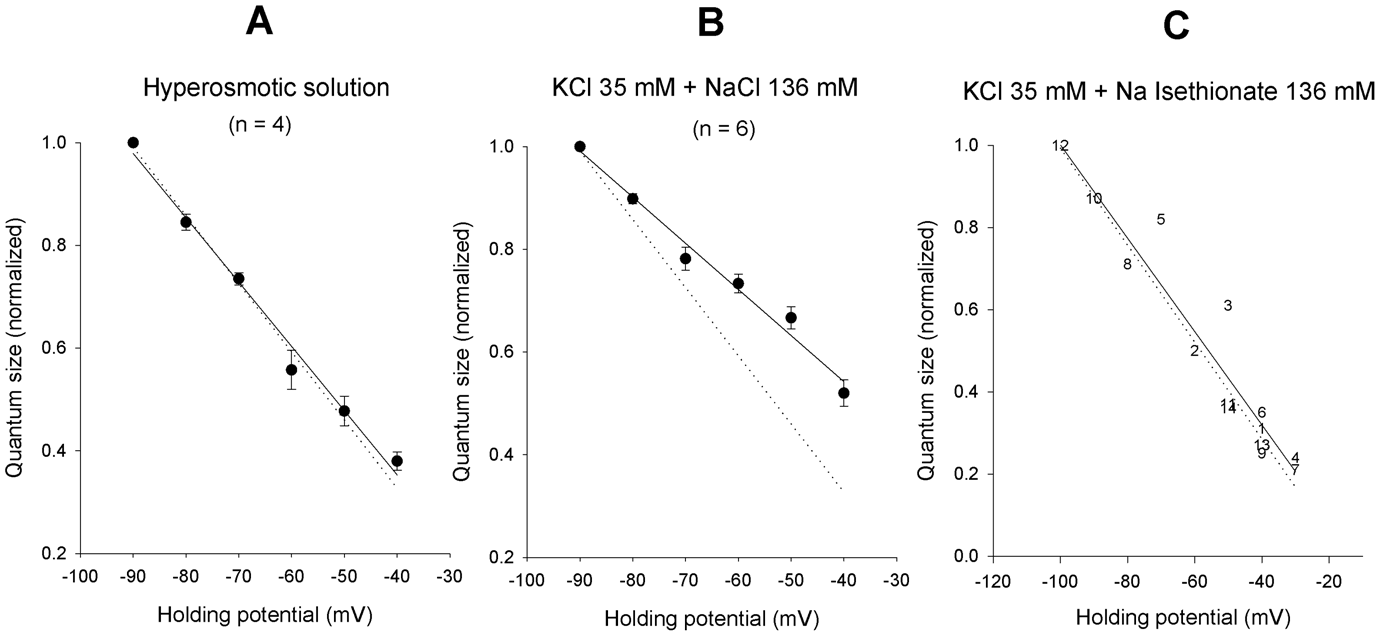
mEPSP I–V curves are influenced by the impermeant chloride ion. (**A**). I–V relationship of mEPSPs recorded, under two-electrode current clamp conditions at different holding potentials in the −40/−90 mV range, in 4 neurons exposed to an external solution made hyperosmotic (718 mosmol/kg) with 0.37 M sucrose. Quantum size was calculated in the different tracings as described in Figure 4B and C, and normalized to the value measured at −90 mV (−90 mV mean value  = 1.6±0.2 mV). The I–V curve of EPSPs (continuous line) was virtually superimposable to the normalized I–V curve of control EPSCs (dotted line; same data, normalized, as in Figure 1B). Hypertonicity does not seem to affect the I–V relation. (**B**). mEPSP I–V relationship (continuous line) evaluated as in (**A**) in a 6-neuron sample exposed to enriched K^+^ solution (−90 mV quantum size  = 1.3±0.3 mV). Control EPSC I–V relationship is shown as in (**A**) (dotted line), for comparison. The different slope of the curves points to a rightward shift of the null potential in high [K^+^]_e_. (**C**). I–V relationship of quantal events recorded in a single neuron at different holding levels while exposed to a K^+^-enriched solution in which 136 mM isethionate had been substituted for an isoosmolar amount of NaCl (data normalized to the −100 mV value  = 3.2 mV). The dotted line shows the control EPSC I–V curve (normalized), for comparison. The general behavior appears to have reverted back to control: note that the cation composition of the bathing solution in (**B**) and (**C**) was the same, despite the large differences in the presumable null potential of the mEPSP. Point numbers indicate the order in which the different test levels were successively imposed.

The accuracy of the present EPSC I–V curves is based on the assumption that a constant amount of ACh is released by each supramaximal preganglionic stimulus. In principle, the assumption is tenable, based on the observation that the ACh volley output and the overall EPSC amplitude and properties remain reasonably constant over time. Nonetheless, the EPSC is intrinsically a compound phenomenon, strictly related to the summed effect of quantal transmitter packets, whose single size is not readily measurable. We tested whether some of the results described under voltage-clamp conditions would be confirmed under current-clamp, considering the single quantal units, the mEPSPs. Current clamp was preferred because of the large intrinsic noise of the two-electrode voltage-clamp technique, which makes the analysis of the small unitary currents inaccurate. Application of solutions made hyperosmotic (with sucrose; final value: 718 mosmol/kg; n = 4) or with increased [K^+^]_o_ (35 mM; n = 6) raised the otherwise negligible mEPSP emission rate. A sequence of mEPSPs was recorded while injecting current so to drive the membrane potential for short periods (20–60 s) at various levels in the −30/−100 mV voltage range, in random sequence and more than once, when possible, in the same experiment, from a holding potential of −50 mV. After each episode, the neuron was returned and maintained at −50 mV for 2 min before applying the subsequent voltage step; the voltage-dependent [Cl^−^]_i_ shifts, which obligatorily accompany membrane potential migrations [16], were thus minimized. Quantal emission rate was constant, independent of the type of stimulation and of the postsynaptic membrane potential level; it was higher in the K^+^-enriched solution (30–65 mEPSP/s) than in hyperosmotic conditions (27–32 mEPSP/s). Typical recordings at two different test potentials are shown in Figure 4A, together with the mEPSP amplitude and interval distributions (Fig. 4B and C). The mean amplitude varied almost linearly with voltage (Fig. 4B a,Ca and Fig. 5), as expected (the passive neuron properties are reasonably constant over this voltage range, especially during short time periods), and the random nature of the presynaptic release mechanism was not affected by the postsynaptic membrane potential (Fig. 4B b and Cb). The current-voltage relations were not fit with Goldman current equations under these conditions, because the size of the mEPSP is not expected to be linear with the conductance, especially as membrane potential approaches the null potential. The comparisons were performed semi-quantitatively by normalizing the values measured in each experiment to the mean mEPSP amplitude at −90 mV and fitting a linear regression curve. The dotted line in each panel of Figure 5 displays the control EPSC I–V curve, similarly normalized, as a visual control.

The normalized I–V curve of mEPSPs obtained in hyperosmotic solution was virtually superimposable on the normalized I–V curve of control EPSCs (Fig. 5A; n = 4). Conversely, when the external potassium was raised to 35 mM, the mEPSP I–V curve (Fig. 5B; n = 6) displayed a reduced slope, indicative of a rightward shift of the null-point (more pronounced than expected solely based on the increase in extracellular K^+^ concentration, which should shift the null point by some 7 mV); in this experiment the sodium complement was kept constant and KCl was simply added to the normal saline, making it slightly hypertonic (this is not expected to modify the synaptic null potential, see above) and increasing the extracellular chloride concentration by about 30 mM, which instead might shift the I–V curve a few mV to the right. By reducing the [Cl^−^]_o_ in the solution enriched with potassium (isoosmotic substitution of 136 mM Na-isethionate for NaCl), the I–V curve moved back towards the control. A typical experiment is illustrated in Figure 5C. The negative shift of the E_ACh_ in low chloride concentration, observed under voltage-clamp conditions, was thus mirrored, at least qualitatively, by the behavior of the mEPSP I–V relationship.

### Simulation of the effects of a variable E_ACh_

**Figure 6:**
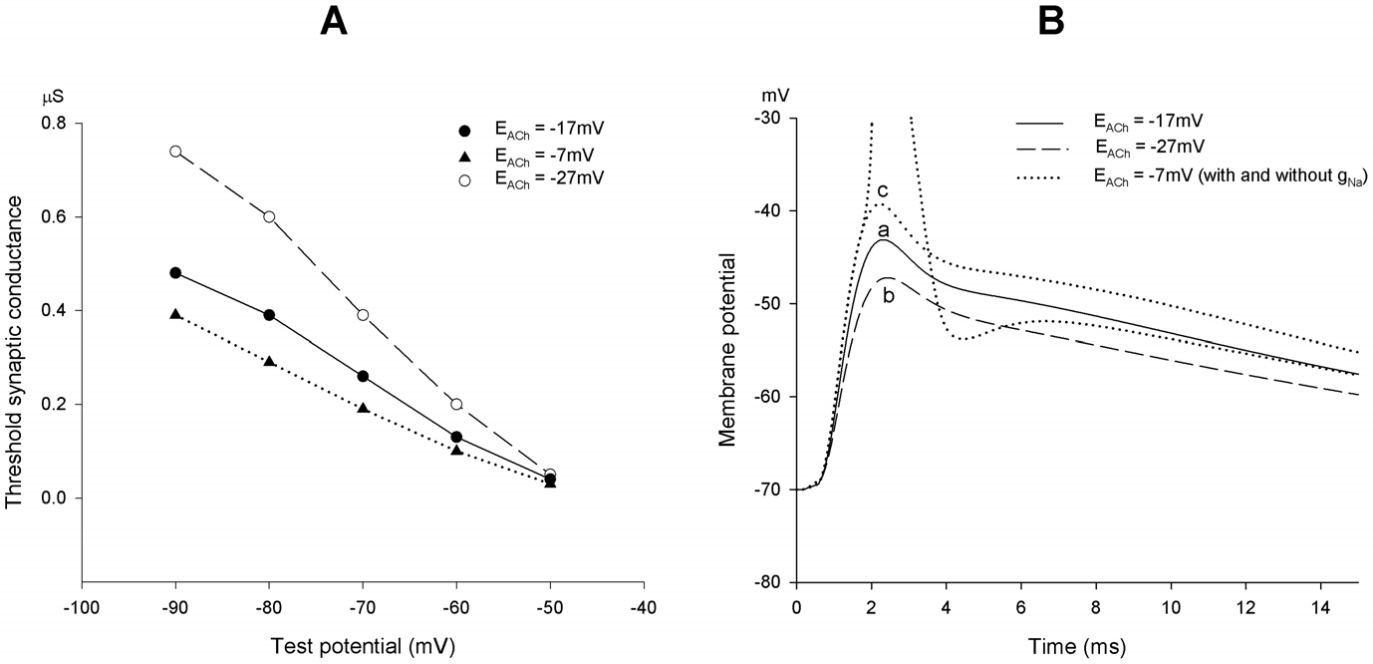
Neuron excitability is regulated by shifts of the EPSC null potential. (**A**). Calculation of the threshold synaptic conductance required to fire the ideal sympathetic neuron held at different membrane potential levels (−50/−90 mV range); ±10 mV changes in E_ACh_ from the normal −17 mV mean value are considered. (**B**). Simulation of the EPSP generated in the model neuron held at −70 mV by a just subthreshold synaptic conductance (0.27 µS), with E_ACh_ = −17 mV (a). The effects of the same value of synaptic conductance are examined with the value of E_ACh_ moved to −27 mV (b) or to −7 mV (c). The synaptic response is larger in (c) and, if active voltage-dependent sodium conductance (g_Na_) is present, the synaptic input results adequate to fire the neuron, otherwise silent.

A computational model has been previously developed for the action potential and, more generally, the electrical behavior of the rat sympathetic neuron, based on a complex system in which five voltage-dependent conductances and the activating synaptic conductance coexist [23], [24]. The individual current components are mathematically described, making the model flexible, in that each of the variables can be controlled and modified. The model was adapted to simulate the effects of the modification of the E_ACh_ on the overall electrical behavior of the neuron. In Figure 6A the computed value of the threshold synaptic conductance required to evoke the action potential (g_syn_*) is represented, with the ideal neuron held at various membrane potential values in the −50/−90 mV range, and assuming a mean E_ACh_ of −17 mV. At each voltage level, a positive-negative 10 mV shift of the synaptic equilibrium potential was simulated and the new g_syn_* values were estimated. A varying driving force of the synaptic current resulted in relevant effects on neuron excitability. For example, a 50% larger g_syn_* was required to fire the neuron held at −70 mV when E_ACh_ moved to −27 mV, while a 26% lower g_syn_* was sufficient to activate the same neuron following E_ACh_ shift to −7 mV. The effect became increasingly larger with increased membrane potential negativity. A complementary simulation is presented in Figure 6B, in which the EPSPs evoked by a constant amount of ACh released onto an ideal sympathetic neuron are computed, considering the null potential migrations of the postsynaptic nicotinic receptors analyzed in Figure 6A. The g_syn_ was arranged to generate an EPSP just close to the firing threshold (Fig. 6B a). Thereafter, the numerical value of g_syn_ was maintained constant, but a 10 mV negative shift of E_ACh_ was considered: the EPSP was reduced in amplitude and the neuron response fell short from threshold (Fig. 6B b). When the E_ACh_ was set at −7 mV, the EPSP produced by the same g_syn_ became sufficient to elicit the action potential (Fig. 6B c). Simulations confirmed that any action on the driving force of the postsynaptic current, at constant presynaptic transmitter release, resulted in relevant readjustment of the neuronal excitability machinery. This process thus represents an additional powerful integrative mechanism of neuron behavior.

## Discussion

The present results suggest that the permeability of the native nicotinic channel at the ganglionic synapse to the physiologically permeant ion species – sodium and potassium – can be modified by relatively simple manipulations, such as membrane voltage history, anionic composition of the extracellular solution or toxin application.

In particular, moving membrane potential from −40 mV to −90 mV produces a 34% decrease in P_K_/P_Na_ ratio, with a 21% decrease in cell synaptic conductance. Conversely, reducing extracellular Cl^–^ concentration from the physiological 154 mM to 18 mM produces a 30–44% increase in P_K_/P_Na_, accompanied by a 20% decrease in synaptic conductance; application of αBgTx produces similar effects, i.e. a 46% increase in P_K_/P_Na_, accompanied by a 10% decrease in conductance. The effects of reduced extracellular chloride and αBgTx on the permeability ratio are mutually occlusive, whereas their effects on permeability are additive.

Although the population of nAChRs at the sympathetic neuron may be comprised of several combinations of receptor subunits, and it cannot be excluded that the composition of the subsynaptic population of receptors may change under particular conditions, it is very unlikely that the procedures here considered may produce such changes. Actually, it appears that the biophysical properties of the channel itself, and in particular its selectivity for the various cations, are influenced by the binding of toxins and the concentration of (impermeant!) chloride ions at the inner and/or outer mouth of the pore. The modified permeability properties of the channel and the ensuing changes in functional null potential result in significant modification of the synaptic current driving force and magnitude, and are able to produce major effects on neuronal excitability.

### The role of membrane potential

In principle, the EPSC I–V relations might be affected by voltage-dependent, slow changes in the concentrations of the cations that carry the synaptic current. This would produce changes in the driving forces across the channel and determine a shift in reversal potential, which would be erroneously attributed to a change in P_K_/P_Na_ ratio. However, cation concentration should change by a factor of 1.5 to shift the null point by 10 mV or more.

As regards [K^+^]_i_ in rat sympathetic ganglia, the subthreshold potassium conductance (g_K_) is virtually constant at about 35 nS per neuron over the −40/−120 mV voltage range [16]; it generates a mean potassium current of about 1.7 nA at −40 mV. Tail-current analysis indicates that this I_K_ intensity has minimal effects on the potassium equilibrium potential, E_K_ (see Figure 2 in Belluzzi et al. [25]), and therefore on intracellular K^+^ concentration. In principle, outward K^+^ current should be lower at −90 mV than at −40 mV; this might produce a slight increase in [K^+^]_i_ with hyperpolarization, and a consequent leftward shift of E_ACh_, which is opposite to the observed shift, illustrated in Figure 1B. Moreover, significant changes in E_K_ result in modification of the ion driving force, which would be readily detected by considering the amplitude of the voltage-activated potassium currents; I–V curves of evoked current were actually insensitive to external anion modification, pointing to small, if any, changes in [K^+^]_i_. Finally, the intracellular potassium activity was measured in rat sympathetic neurons by using ion-sensitive microelectrodes: it was found to be unaffected by membrane potential movements in the −40/−80 mV range (Figure 1 in Ballanyi and Grafe [26]).

As concerns [Na^+^]_i_, hyperpolarization-induced elevation was described in pyramidal neurons of mouse hippocampus [27] and dopamine cells of the substantia nigra [28]. In both cases, however, [Na^+^]_i_ changes were blocked by cesium, and were considered to be due to Na^+^ influx through the non selective I_h_ cation channel. In the sympathetic neuron, voltage-dependent sodium channels are closed above −40 mV [23] and subthreshold membrane currents are generated exclusively by a variable mix of chloride and potassium conductances; when these are blocked no other conductances can be detected, except the small voltage-independent leakage conductance (a mean of about 14 nS per neuron [16]). On the other hand, [Na^+^]_i_ should increase by a factor of 1.5 to shift the null point by 10 mV; such a high concentration is reached in neurons only in the presence of blockers of active sodium pumping. Direct evidence of internal ionic stability arises from the observation that GABA-induced membrane depolarization or hyperpolarization in rat sympathetic neurons did not change the intracellular sodium activity, measured with ion-selective microelectrodes [26].

Shifts in the potassium and sodium ion gradients were thus quite unlikely explanations for the changes in EPSC I–V curves observed following modifications in holding potential.

Direct effects of holding membrane potential on the permeability properties of the channel might well occur. The main problem with this interpretation of the data is related to the very slow time course (minutes) of these changes. An intriguing aspect is that chloride ions redistribute following membrane potential migrations, and this equilibration similarly requires tens of seconds to minutes. Chloride concentration in the rat sympathetic neuron was found to be systematically larger than predicted by a passive equilibrium, thereby generating a slowly adapting, voltage-dependent chloride battery, which plays a pivotal role in controlling the membrane potential in the subthreshold voltage range, through a voltage- and time-dependent chloride conductance [16]. In particular, the −40/−90 mV membrane potential change is expected to be accompanied by a [Cl^−^]_i_ decrease from about 39 to 13 mM at steady-state ([16], Figure 3B), and vice-versa upon returning to the initial −40 mV holding level. At present, the threefold change in intracellular chloride concentration appears to be the most relevant ionic change.

The effect of holding potential on the channel properties might therefore be indirect, and mediated by a change in intracellular chloride concentration. This idea is strongly supported by the observation that the I–V curves become insensitive to holding potential when [Cl^−^]_o_ is reduced by about tenfold (18 mM), a condition under which chloride redistribution is largely impaired and intracellular chloride concentration is very unlikely to undergo massive fluctuations in response to migrations of the membrane potential.

### The action of αBgTx

Alpha-7 homomeric channels have not been detected in rat sympathetic neurons [29], [30]. These neurons, however, express an αBgTx-sensitive nAChR which is likely to incorporate the α7 subunit: 100% of fresh-cultured neurons of the rat SCG are actually stained with antibodies to α7 [31] and two classes of αBgTx-binding sites have been detected in dissociated SCG neurons [32]. Previous studies have shown, by utilizing rapid agonist application on dissociated rat SCG neurons at constant membrane potential, that αBgTx was either without effect on any current [33], or able to block up to 80% [31] or 40% [32] of the ACh induced currents. In none of those studies was it possible to discern whether the toxin acted on synaptic or extrasynaptic receptors. In the present study the specific biophysical effect of the toxin on subsynaptic receptors has been addressed in mature, intact sympathetic neurons. We show that αBgTx significantly decreases the synaptic conductance, slightly reduces the mean open time of the synaptic nicotinic channel and sustains a negative shift of the E_ACh_ by altering the P_K_/P_Na_ ratio.

The difference current, abolished by αBgTx application, displays a strongly positive null potential. This might arise from a change in the permeability properties of the channel, or from the block of a specific current component (a Ca^2+^ current would be suggested by the positive equilibrium potential).

A negative extrapolated null potential was estimated for α7 homo-oligomeric channels expressed in Xenopus oocytes (−28 mV, see Couturier et al. [34]; −17 mV, Forster and Bertrand [35]). Massive changes in [Ca^2+^]_o_ were shown to produce negligible effects on the nAChR null potential at the sympathetic neuron [9]. The experiments here reported indicate that blockers of the α7-containing receptors (F3 and MLA), with presumably higher selectivity, are unable to mimic the effect of αBgTx on E_ACh_. Thus, it is most unlikely that αBgTx simply blocks a subset of calcium-permeable synaptic channels. The amplitude decrease of the EPSC measured at a single membrane potential level might, in fact, be related to changes of both null potential and synaptic current kinetics, which contribute to various extents in shaping the overall EPSC I–V curve. In order to clarify the question, analysis of native nAChRs should be extended at the single channel level; unfortunately, patch clamp of unequivocally subsynaptic receptors in the intact sympathetic neuron is technically unfeasible.

All considered, a likely mechanism of αBgTx action appears to involve a change in the selectivity of the nicotinic channel, which would increase its ability to carry potassium ions. If this were the case, interference should be observed with other treatments that affect the channel selectivity. This aspect is considered below.

### The effect of chloride and the interaction with αBgTx

The most relevant, and unexpected, observation here reported is that changes in external, and possibly internal, non-permeant chloride ions regulate the behavior of the nicotinic channel. Drastic reduction of [Cl^−^]_o_ results in synaptic effects that are similar, qualitatively and quantitatively, to those sustained by αBgTx; namely, the apparent synaptic conductance and the mean channel open time are reduced, the channel selectivity is equally modified, and the missing fraction of the synaptic macrocurrent exhibits a strong positive equilibrium potential. Calcium-related effects can be readily ruled out in reduced [Cl^−^]_o_ experiments: 1) changes in subunit composition (differentially calcium-permeable) are unlikely here, 2) isethionate can bind calcium ions, but benzenesulphonate does not; 3) large modifications in [Ca^2+^]_o_ have negligible effects on the E_ACh_.

Once more, a change in cation permeability ratio, and in particular a decreased Na^+^ *vs*. K^+^ permeability in low [Cl^−^]_o_, is the most likely interpretation. The toxin and chloride reduction mutually occlude their effects on channel permeability, independent of the order of application, pointing to the involvement of a common mechanism.

A few studies have examined the functional role of chloride ions as regulators of biological macromolecules, typically via allosteric mechanisms. The activation of kainate receptors requires the presence of chloride in the extracellular solution [36]; glycine receptors gating was found to be profoundly affected by high chloride [37]. These analyses, however, mainly focussed on single receptor kinetics and were not accompanied by the study of the permeability properties of the single channel. This type of information on the nicotinic channel is lacking in the literature. From our electrophysiological data it is impossible to tell whether and how occlusion between αBgTx and chloride binding occurs, whether the sites of action are physically the same and which might be the molecular counterparts.

### The ganglionic selectivity filter can dynamically change

The selectivity characteristics of the nicotinic channel at the ganglionic synapse have long been studied [38], [18]. The mix of sodium and potassium conductances and their ratio, which mould the synaptic cationic current, are considered to be kept constant, constrained by the physical structure of the channel pore [39],[40]. More generally, no experimental evidence has so far suggested any time-dependent dynamic changes in the selectivity of the nicotinic filter, or of the 5-HT_3_ receptor, a ligand-gated ion channel that is a cation-selective member of the nicotinic receptor family [41], [42]. In our preparation, g_syn_ and E_ACh_ usually are stable over periods of tens of minutes under steady-state conditions, at constant holding potential and with appropriate modes of presynaptic stimulation (see, for example, Figure 1 in Sacchi et al. [43], and unpublished observations).

Early denervation [9], the history of membrane potential, a reduced external (and possibly internal) chloride concentration and αBgTx (present data) reversibly modulate postsynaptic ACh response amplitude and decay kinetics, with a clear-cut shift in the nicotinic channel P_K_/P_Na_ ratio, estimated by using the Goldman current equation to fit the entire I–V relationship of the EPSC. The results of all these analyses on native, unequivocally and exclusively subsynaptic nicotinic channels are summarized in Table 2. Reduced [Cl^−^]_o_ and αBgTx increased the calculated channel selectivity for K^+^ with respect to Na^+^ ions, while membrane hyperpolarization and presynaptic denervation resulted in a loss of the relative selectivity of the channel, which became equally permeable to both ions.

**Table 2:** Effect of different treatments on the permeation properties of the nicotinic channel at the ganglionic synapse.

Treatment	P_K_ (fl/s)	P_Na_ (fl/s)	P_K_/P_Na_	
[Ca^2+^]_o_ = 2 mM (n = 5)	0.619	0.423	1.46±0.12	Sacchi et al., unpublished
[Ca^2+^]_o_ = 5 mM	0.874	0.609	1.43±0.13	
Control (n = 6)	1.896	1.121	1.69±0.19	This paper
F3^a^ 10 µM	1.242	0.730	1.68±0.13	
Holding potential = −40 mV (n = 10)	0.984	0.701	1.40±0.13	This paper
Holding potential = −90 mV	0.582	0.632	0.92±0.10	
[Cl^−^]_o_ = 154 mM (n = 10)	2.162	1.361	1.57±0.09	This paper
[Cl^−^]_o_ = 18 mM	2.229	0.977	2.26±0.08	
[Cl^−^]_o_ = 154 mM (n = 6)	1.903	1.314	1.52±0.11	This paper
αBgTx 200 nM, in [Cl^−^]_o_ = 154 mM	2.236	1.024	2.22±0.13	
αBgTx + isethionate, [Cl^−^]_o_ = 18 mM	1.821	0.762	2.38±0.07	
Control (n = 33)			1.56^b^	[9]
Denervated (n = 26)			1.07^b^	

Single treatments were tested on the same group of neurons, except in the case of denervation.

a F3 is a 4-oxystilbene derivate with a high degree of antagonist selectivity for neuronal nicotinic αBgTx receptors containing the α7 subunit.

b Calculated by fitting the Goldman current equation to mean I–V data.

Values are means ± SEM.

From a physiological point of view, it is worth noting that the selectivity changes of the synaptic channels and the ongoing neuronal activity (and the associated membrane potential migrations) reciprocally influence each-other. The flexible permeability of the synaptic channels therefore constitutes an additional aspect in neuronal regulation and plasticity, as it results in changes in the driving force of the synaptic current, and consequently in time- and activity-dependent variations in the postsynaptic response to an invariant presynaptic input.

### Possible artifacts and alternative interpretations

The evidence in favor of the selectivity hypothesis might suffer from systematic errors in electrophysiological measurements or might be challenged by alternative biophysical interpretations.

As previously discussed, a variable junction potential at the electrodes might bias, in principle, the present voltage values when external chloride concentration is changed or its internal concentration changes due to a shift in membrane potential (Sacchi et al., 1999 [15]). Liquid junction potentials have been computed, according to Henderson's approximation [44], for the microelectrode and agar bridge in the various solutions encountered in these experiments; the resulting corrections are reported in Text S1: they never exceed a 2.5 mV magnitude; when the corrections are applied, the P_K_/P_Na_ ratios slightly increase (+0.01 to +0.18). However, these corrections were too small to interfere with our conclusions.

A series of experimental test argue against artifacts linked to junction potentials:

1) the liquid junction potential, measured with the 3MKCl agar-bridge and the 3MKCl microelectrode broken-tip technique, was found to be small and stable; 2) the I–V curves for potassium currents in normal or reduced external chloride concentration were overlapping (the voltage sensor of the potassium channel is very sensitive so that any voltage shift around −15 mV would drastically modify the current amplitude); 3) identical external chloride modifications markedly affected E_ACh_ in the absence of αBgTx, but minimally in the presence of the toxin; 4) bathing in low chloride (18 mM) + isethionate solutions is supposed to be the most challenging treatment in terms of liquid junction potentials, as the agar bridge can be contaminated by the bathing solution and produce a relevant change in liquid junction potential upon returning to normal chloride containing (154 mM) solution; however, estimates of E_ACh_ and P_K_/P_Na_ ratio displayed full reversibility, arguing against marked artifacts in potential measurement.

As concerns the battery of the permeant cations, the potassium and sodium internal concentration following neuron hyperpolarization was measured in the sympathetic neuron, and was found unchanged over the voltage range tested here [26]. Over the same voltage range, the voltage-dependent sodium channels are closed [23] and the sodium-dependent co-transports do not significantly contribute to control baseline intracellular sodium, at least in cultured hippocampal neurons [45]. The potassium current evoked by depolarization are of constant amplitude when external anion composition is modified, ruling out any significant shift in the potassium driving force. Finally, either the sodium or the potassium internal concentrations should decrease by an unrealistic amount during hyperpolarization (contrary to the direction expected from the electric field), if these ionic gradient changes were underlying the E_ACh_ shift.

The hypothesis that multiple channel types, with different selectivity properties, might be differently modulated by the treatments, is inaccessible to direct biophysical testing, especially if only subsynaptic channels are to be considered. On the other hand there is no notion that nicotinic channels with different alpha-beta subunit compositions might exhibit different ionic selectivities in native neurons, except for those containing the α7 subunit. The possible participation of the latter receptors in contaminating the pure Na^+^/K^+^ fluxes with a calcium component, however, has been ruled out by applying the selective α7 antagonists.

Apart from intracellular bulk cation concentrations, one may wonder whether the AChR channel I–V curve might be affected by local heterogeneities in cation concentrations. In principle, channel selectivity might appear to change due to altered local cation concentration at the mouth of the pore, rather than to an intrinsic change in pore selectivity filter. If the channel presents a vestibule at the pore access, and chloride is the major mobile anion which can access the vestibule, a Donnan equilibrium ensues between the vestibule and bulk solution (both intra- and extracellularly). Assuming that a difference exists between the concentration of fixed negative charges in the vestibule and anions in solution that cannot access the vestibule, a difference *ΔCl* ^–^ is expected to occur between [Cl^−^]_v_ and [Cl^−^]_s_, where the “v” and “s” subscripts respectively refer to the vestibule and the bulk solution. Thus, a Donnan equilibrium should ensue, with 
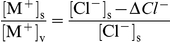
, where M^+^ indicates monovalent cations, and a change in [Cl^−^]_s_ should produce a change in cation concentration in the vestibule. This would affect the driving force on the cations and be reflected in the conductance ratio of the pore (which is determined by both the permeability ratio and the concentration ratio for sodium and potassium). However, it is easily shown that a corresponding Donnan's potential 
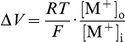
 will arise under these conditions at the vestibule-solution interface, which exactly offsets the altered vestibular cation concentration in controlling cation flux. Thus, effects of chloride concentration, either on the intracellular or the extracellular side, would not be able to affect the measured null potential of the channel and the computed P_K_/P_Na_ ratio. By similar reasoning, possible effects of chloride ions on the profiles of transmembrane electrical field or local cation concentrations, and local screening effects (which are quite unlikely for a monovalent ion), would affect to the same extent the two permeating cations and would in no case be able (due to simple thermodynamic consideration) to introduce any asymmetry in the inward vs. outward permeation. On the other hand, chloride ions might affect the permeability ratio for differently sized cations by binding allosterically to specific sites on the channel protein (not necessarily within the pore) or by binding to fixed charges within the pore, thereby affecting the minimum pore diameter or the dehydration energy for the permeating cations.

We have no direct evidence for a structural change of the nicotinic channel that might affect its selectivity. It should be stressed, however, that while each single source of artifact or biophysical interpretation (listed above, or others) could explain a single circumstantial result, none of them *alone* is able to give an unifying view of the complex of toxin and voltage- or anionic-dependent effects on ACh null potential.

### Does a common mechanism underlie these examples of nicotinic channel modulation?

A new role for chloride ions is envisaged here. It might represent a common key to understand findings obtained under otherwise heterogeneous experimental conditions. Steady membrane potential shifts modify [Cl^−^]_i_, while the concentrations of the other major ions, Na^+^ and K^+^, are presumably hardly affected. External chloride modification, on the other hand, is able to evoke effects similar to those of αBgTx, and the two actions are partly overlapping and mutually occlusive. The channel cation selectivity is reduced by increased chloride gradient (membrane hyperpolarization), while it is increased, moving towards a channel preferentially permeable for potassium, when the chloride gradient is reduced. The EPSC decay accelerates when either external or internal chloride is reduced, suggesting that anions modulated nicotinic receptors from both sides of the plasma membrane; this effect was apparently insensitive to the momentary membrane potential.

## Supporting Information

Text S1 Liquid Junction Potentials.
(DOC)
