# Supplementary material for: Changes in Cationic Selectivity of the Nicotinic Channel at the Rat Ganglionic Synapse: A Role for Chloride Ions?
Source: PLoS One. 2011 Feb 25;6(2):e17318. doi: 10.1371/journal.pone.0017318 (PMC3045433; doi:10.1371/journal.pone.0017318)
Supplement: Text S1 — Liquid Junction Potentials. (DOC) [file pone.0017318.s001.doc]

**SUPPORTING INFORMATION**

**SUPPLEMENTAL TEXT S-1**

Liquid Junction Potentials

In order to evaluate whether – and to what extent – the observed changes in I-V curves, and consequently in P_K_/P_Na_ and E_ACh_ estimates, were affected by changes introduced by the experimental procedures on membrane potential measurements, the liquid junction potentials at the voltage microelectrode and reference agar bridge were systematically computed, using the standard approximation by Henderson [44].

The change in liquid junction potential at the electrode-solution interface, upon impalement of the cell, can be estimated, under the extra- and intracellular ionic composition in control condition, to ‑2.46 mV. This correction should be applied to all measurements.

Changes in intracellular and/or extracellular chloride ions are expected to introduce shifts in such diffusion potentials, respectively, at the microelectrode and reference electrode interfaces.

Chloride concentration in the rat sympathetic neuron was found to slowly adapt to membrane potential. A ‑40 to −90 mV membrane potential change is accompanied by a [Cl^−^]_i_ decrease from about 40 to 13 mM. The threefold change in intracellular chloride concentration is expected to introduce a change in intracellular electrode liquid junction potential from −2.46 mV to −2.15 mV (4 M K-acetate electrode; the values for 3 M KCl, used in two double-check experiments, would be from −3.12 to −3.67 mV). So, a 0.3 mV error may be present in our estimates of E_Ach_ shifts following membrane potential migrations from −40 mV to −90 mV and back. The corrected values, for the measurements reported in the Results section, are reported in Table S-1.

Substituting 136 mM isethionate for an equal amount of chloride in the bath solution might similarly introduce small displacements in liquid junction potantial at the bath / reference agar bridge electrode interface. In particular, the computed liquid junction potentials would move by −2.50 mV (intracellular minus reference = from –2.46 mV to +0.04 mV). The values of potassium/sodium permeability ratio and the corresponding E_ACh_ shift, after correction for diffusion potential at the reference electrode-bath interface are also reported in Table S-1.

| nominal  V_H_ (mV) | corrected  V_H_ (mV) | [Cl^–^]_i_  (mM) | [Cl^–^]_o_  (mM) | P_K+_/P_Na+_ | Δ P_K+_/P_Na+_ (%) | E_ACh_  (mV) | Δ E_ACh_ (mV) |
| --- | --- | --- | --- | --- | --- | --- | --- |
| – 40 | – 42.5 | 40 | 154 | 1.56 | – 35% | – 17.7 | + 9.9 |
| – 90 | – 92.2 | 13 | 154 | 1.02 |  | – 7.8 |  |
| – 40 | – 42.5 | 40 | 154 | 1.75 | + 29 % | – 20.1 | – 5.8 |
| – 40 | – 40.0 | 40 | 18 | 2.26 |  | – 25.9 |  |

Notice that upon changing the bathing solution two phenomena ensue: (i) a new diffusion potential arises, if osmolarities have been changed or ions with different mobility have been introduced and (ii) the solution within the agar bridge may slowly get contaminated by the bathing solution.

The liquid junction potential, which is well approximated by Henderson's formula, can be analytically considered in terms of Planck's equation. For each ion species (*i*), the flux (*J*) is defined by: $J_{i}=-u_{i}RT\frac{dc_{i}(l)}{dl}-z_{i}u_{i}c_{i}(l)F\frac{d\psi(l)}{dl}\begin{matrix} & 0\leq l\leq L \end{matrix}$ (1)

where *u* and *z* respectively indicate ion mobility and valence, *F* and *R* are Faraday’s and Boltzmann’s constants, *T* is absolute temperature (°K), *c* and $\psi$ indicate concentration and electrical potential as functions of location (*l*) along the diffusion layer.

Under almost steady state conditions (small time-dependent changes in the concentration and potential profiles), the fluxes can be considered constant with respect to position, $J_{i}=\text{const}(l)$, which yields: $J_{i}=u_{i}RT\frac{c_{i}(0)\mathbf{e}^{z_{i}F\psi(0)/RT}-c_{i}(L)\mathbf{e}^{z_{i}F\psi(L)/RT}}{\int_{0}^{L} \mathbf{e}^{z_{i}F\psi(l)/RT}dl}$ (2)

The liquid junction potential is the zero-current solution of the set of equations (2) for all ion species: $\sum z_{i}J_{i}=\sum z_{i}u_{i}RT\frac{c_{i}(0)\mathbf{e}^{z_{i}F\psi(0)/RT}-c_{i}(L)\mathbf{e}^{z_{i}F\psi(L)/RT}}{\int_{0}^{L} \mathbf{e}^{z_{i}F\psi(l)/RT}dl}=0$ (3)

As regards the slow contamination of the agar bridge in electrophysiological experiments, and possible resulting drifts in liquid junction potential, it may be observed that, once a function $\psi(l)$is found which solves eqn. (3), if the boundary conditions, *c*(0) and *c*(*L*), are not changed, then any function $\xi(h)=\psi(l)\begin{matrix} & \left\{\begin{matrix} h=b\cdot l \\ 0\leq h\leq b\cdot L \end{matrix}\right. \end{matrix}$ will also solve eqn.(3):

$\sum z_{i}J_{i}=\sum z_{i}u_{i}RT\frac{c_{i}(0)\mathbf{e}^{z_{i}F\xi(0)/RT}-c_{i}(L)\mathbf{e}^{z_{i}F\xi(bL)/RT}}{\int_{0}^{bL} \mathbf{e}^{z_{i}F\xi(h)/RT}dh}$ = $\sum z_{i}u_{i}RT\frac{c_{i}(0)\mathbf{e}^{z_{i}F\psi(0)/RT}-c_{i}(L)\mathbf{e}^{z_{i}F\psi(L)/RT}}{b\cdot\int_{0}^{L} \mathbf{e}^{z_{i}F\psi(l)/RT}dl}$

This indicates that during time, as the thickness of the diffusion layer increases, the electrical field and concentration profiles will preserve the same shape, and will simply get stretched by an increasing factor along the diffusion layer, while all fluxes will be decreased by the same factor.

Thus, the diffusion potential is expected to depend on the ionic compositions of the two limiting solutions but not on the thickness of the diffusion layer (or time).

The above considerations only assume a location-independent flow for each ion species; this condition is only violated if the concentration profiles change significantly and rapidly enough, which usually occurs only just after establishing the contact between the two solutions.

Contamination of the agar bridge, during our experiments of isethionate substitution for chloride, is not therefore expected to introduce significant time-dependent drifts in measurements of membrane potential. However, such contamination would certainly affect the liquid junction potential one would observe upon switching to a new bath solution with a different ionic composition or back to the control solution.

As reported in the text, the changes observed in isethionate were fully reversible upon returning to the control condition, which suggests that agar bridge contamination and resulting artifacts related to liquid junction potentials were not significant in our experiments.
